# Cation−π Interactions in Biomolecular Contexts by Neutron Scattering and Molecular Dynamics: A Case Study of the Tetramethylammonium Cation

**DOI:** 10.1021/acs.jpcb.5c02001

**Published:** 2025-06-27

**Authors:** Matej Cervenka, Brennon L. Shanks, Philip E. Mason, Pavel Jungwirth

**Affiliations:** Institute of Organic Chemistry and Biochemistry of the Czech Academy of Sciences, Flemingovo nám. 2, Prague 6 166 10, Czech Republic

## Abstract

Cation−π interactions involving the tetramethylammonium motif are prevalent in biological systems, playing crucial roles in membrane protein function, DNA expression regulation, and protein folding. However, accurately modeling cation−π interactions where electronic polarization plays a critical role is computationally challenging, especially in large biomolecular systems. This study implements a physically justified electronic continuum correction (ECC) to the CHARMM36 force field, scaling ionic charges by a factor of 0.75 to effectively account for electronic polarization without additional computational overhead. This approach, while not specifically designed for cation−π interactions, is shown here to significantly improve predictions of the structure of an aqueous tetramethylammonium–pyridine complex as compared to neutron diffraction data. This result, together with computational predictions for the structure of the aqueous tetramethylammonium–phenol complex, underscores the potential of ECC as a versatile method to improve the description of cation−π interactions in biomolecular simulations.

## Graphical abstract

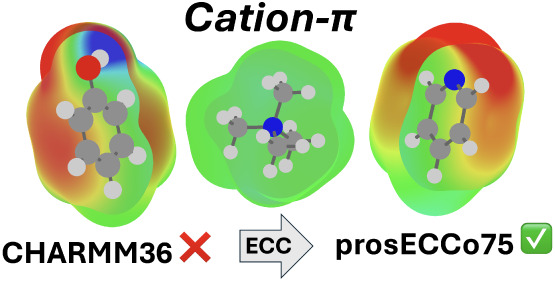

## Introduction

Tetramethylammonium (TMA, N­(Me)_4_^+^) is a common cationic motif in biomolecular systems, notably present as choline in the polar headgroups of phosphatidylcholine lipids. Moreover, the TMA motif is important for choline-binding proteins and is integral to DNA expression regulation through the methylation of lysine residues in histones.^1^ Interestingly, in aqueous environments, TMA does not predominantly associate with anionic species but rather with hydrophobic aromatic molecules like benzene.^2^ This suggests that within proteins, TMA likely interacts with aromatic side chains of tryptophan, tyrosine, or phenylalanine, often forming complex structures such as aromatic cages. These configurations highlight TMA’s significant role in cation−π interactions.^3,4^ Researchers have extensively studied the fundamental nature of cation−π interactions, demonstrating that they are primarily electrostatic, driven by the interaction between the aromatic system’s quadrupole moment and the cation.^5^ Additionally, polarization forces are important, as the cation induces a dipole in the π-electron cloud.^6^

Significant efforts have been made to capture cation−π interactions in standard nonpolarizable force field molecular dynamics (FFMD) simulations. While often qualitatively describing the interaction geometry, these methods fail to achieve quantitative accuracy.^7–10^ Interaction energies are typically underestimated compared to quantum mechanical calculations, making FFMD simulations hardly suitable for tasks like identifying drug candidates reliant on cation−π binding. Khan et al. aimed at addressing this issue by optimizing the 12–6 Lennard-Jones (LJ) potential parameters in the CHARMM36 additive force field.^7^ Similarly, Turupcu et al. emphasized the significance of induction effects by introducing a 1/*r* ^4^ term, resulting in the 12–6–4 LJ formulation within the OPLS-AA force field.^8^ Felder et al. proposed that nonpolarizable force fields can effectively describe cation−π interactions via suitable adjustments to the distribution of partial charges.^10^ However, modifying the LJ potential and partial charges is challenging, as determining optimal parameters and justifying changes to the charges on both the cation and the aromatic molecule often lack a clear physical basis. Finally, explicit polarization methods, such as Drude polarizable force fields, could improve the accuracy of cation−π interaction modeling. Drude polarizable models have become significantly more efficient, with computational overhead down to approximately 2.6-fold compared to additive force fields.^11^

Given the current landscape of challenges associated with modeling cation−π interactions, we have started exploring methods that can account for polarization effects without adding computational cost by implementing the ECC approach.^12,13^ ECC effectively accounts for electronic polarization in a mean-field way by scaling the (partial) charges on the (molecular) ions by the reciprocal square root of the high-frequency dielectric constant. Historically, nonpolarizable force fields such as CHARMM attempted to account for electronic polarization effects empirically: partial charges for neutral molecules were fitted to water-interaction energies that had first been multiplied by 1.16 (to match the TIP3P dimer), yielding dipole moments larger than their gas-phase values, whereas ionic groups retained full charges. It should be noted, however, that this ad hoc tuning is distinct from more rigorous frameworks such as the ECC and related “halfway-charge” methods, which apply uniform, dielectric-derived scaling of charges or dipoles to all species.^9,14–16^ Our resulting force field incorporating implicit electronic polarizability through charge scaling of ionic moieties, denoted as prosECCo75,^13^ has demonstrated its ability to refine ion–ion interactions in biological contexts effectively. In TMA iodide solutions, ECC-type approaches have also been shown to improve predictions of interfacial properties.^17^ Here, we demonstrate that this approach also has the potential to improve the quantitative accuracy of cation−π interactions while maintaining computational efficiency, making it well-suited for large-scale simulations.

As an experimental complement to FFMD simulation, neutron diffraction with isotopic substitution (NDIS) can provide invaluable insight into structural correlations in aqueous solutions. By leveraging the distinct scattering properties of isotopes, NDIS isolates specific atomic interactions with minimal perturbation of the system. This is achieved by preparing isotopically substituted yet chemically identical solutions and subtracting their scattering profiles to isolate contributions from the substituted nucleus, thus enabling subangstrom resolution.^18–26^ This study integrates FFMD and NDIS to investigate TMA–pyridine cation−π interactions. The high solubility of pyridine in water makes NDIS experiments feasible, with the potential to establish benchmark data not only for the present case but also for modeling biologically relevant cation−π interactions involving TMA with other aromatic molecules, such as phenol or indole.

## Methods

### Neutron Scattering Measurements

NDIS measurements were conducted at 23 °C using the D4C diffractometer at the nuclear reactor of the Institut Laue-Langevin in Grenoble, France.^27,28^ Samples were loaded into a cylindrical null-scattering titanium–zirconium cell with identical geometry for all measurements. The cell had a sample diameter of 5.0 mm, a wall thickness of 0.75 mm, and a beam height of 24 mm. Neutrons with a wavelength of 0.4985 Å were used. Four chemically identical solutions containing 2 m TMACl and 2 m pyridine in water were prepared, differing only in H/D isotopic substitution on the TMA (h_12_-TMA and d_12_-TMA) and the solvent (H_2_O or D_2_O). Diffraction patterns (Figure 1) were recorded for approximately 2 h for each D_2_O solution and 4 h for each H_2_O solution. The collected data were corrected for multiple scattering and absorption effects and normalized to a standard vanadium scatterer.^29^ The total correlation between nonexchangeable hydrogen atoms on TMA and other atomic species in the system can be obtained via the first-order difference functions, $\Delta S_{H_{\mathrm{TMA}}}^{X,D_{2}O}\left(Q\right)$ and $\Delta S_{H_{\mathrm{TMA}}}^{X,H_{2}O}\left(Q\right)$ (Figure 1, eqs 1 and 2). These functions essentially represent a weighted sum of the contributions from the structural correlations involving the H/D atoms of TMA. They are, respectively, defined as (in units of mbarns).

1 ΔSHTMAX,D2O(Q)=73.4·SHTMAHW(Q)−3.7·SHTMAHPy(Q)+31.9·SHTMAO(Q)+11.9·SHTMAC(Q)+3.7·SHTMAN(Q)+1.9·SHTMACl(Q)+3.5·SHTMAHTMA(Q)−122.6

2 ΔSHTMAX,H2O(Q)=−41.1·SHTMAHW(Q)−3.7·SHTMAHPy(Q)+31.9·SHTMAO(Q)+11.9·SHTMAC(Q)+3.7·SHTMAN(Q)+1.9·SHTMACl(Q)+3.5·SHTMAHTMA(Q)−8.0

**Figure 1:**
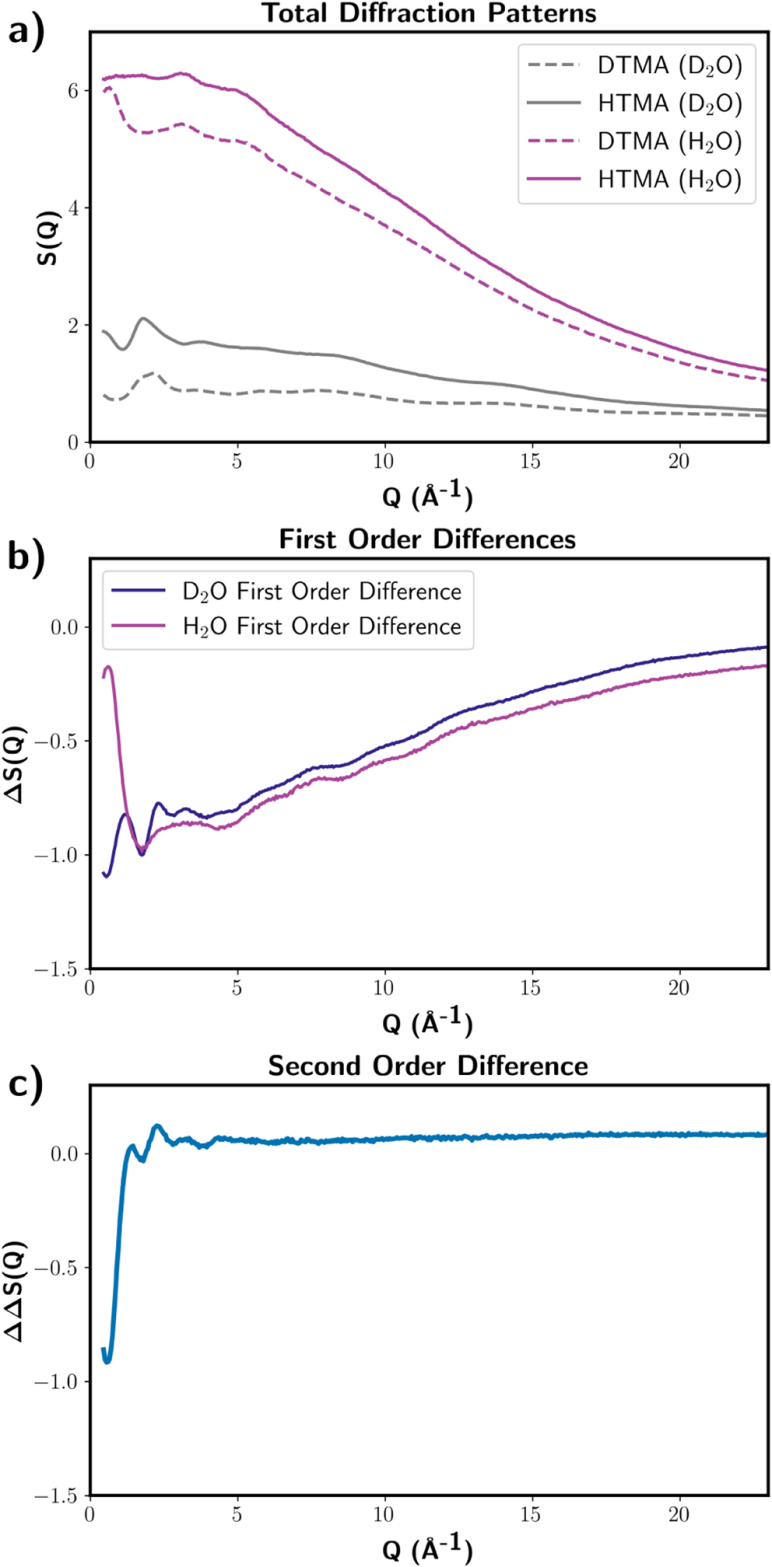
(a) Total diffraction patterns for H_2_O solutions of d12-TMACl and h12-TMACl and D_2_O solutions of d12-TMACl and h12-TMACl: all solutions contain 2 m of pyridine. (b) First-order differences $\Delta S_{H_{2}O}^{X}\left(Q\right)$ obtained by the difference of the two diffraction patterns shown in (a) for H_2_O as a solvent and $\Delta S_{D_{2}O}^{X}\left(Q\right)$ obtained by the difference of the two diffraction patterns shown in (a) for D_2_O as a solvent. (c) Second-order difference $114.5\cdot \left(S_{H_{\mathrm{TMA}}H_{W}}\left(Q\right)-1\right)$ , obtained through the difference of the two first-order differences shown in (b).

Prefactors were calculated using atomic concentrations and neutron scattering lengths of the different elements in the system, following standard literature methods.^30^

The difference between eqs 1 and 2 gives the second-order difference function, ΔΔ*S*(*Q*) (Figure 1c and 3), which provides information on the specific correlation between nonexchangeable hydrogen atoms on TMA and hydrogen atoms in water. This second-order difference is defined as

3 $$\Delta \Delta S\left(Q\right)=\Delta S_{H_{\mathrm{TMA}}}^{X,D_{2}O}\left(Q\right)-\Delta S_{H_{\mathrm{TMA}}}^{X,H_{2}O}\left(Q\right)=114.6\cdot \left(S_{H_{\mathrm{TMA}}H_{W}}\left(Q\right)-1\right)$$

This function serves as a valuable internal consistency check, verifying the accuracy of the solutions as well as the multiple scattering and absorption corrections applied to the data. Due to the significant inelastic scattering of ^1^H and the Placzek effect,^19^ samples containing hydrogen always exhibit a strong background. The higher the atomic concentration of ^1^H, the more pronounced this effect becomes, as observed in light water samples (e.g., Figure 1a). For heavy water samples, this effect is substantially reduced (Figure 1a). The extent of inelastic scattering is primarily determined by the density of ^1^H nuclei, so the first-order differences (Figure 1b) should contain the same Placzek background. If the resulting second-order difference is constant, as shown in Figure 1c, we can conclude that there is no detectable background, indicating that the four solutions were prepared with identical chemical compositions.

Although NDIS can, in principle, measure correlations between any pair of substitutable hydrogens, the feasibility is strongly dependent on sample contrast, which is largely determined by the product of the atomic concentrations. Consequently, H_W_–H_W_ measurements are relatively straightforward, whereas H_W_–H_TMA_ and especially H_TMA_–H_Py_ are more challenging. Notably, the H_TMA_–H_Py_ correlation would provide the most direct insight into cation−π interactions, yet these data were not acquired in earlier experiments. Because neutron beamtime is a scarce resource, the H_TMA_–H_W_ measurement, although less sensitive to cation−π effects, remains the primary experimental data set available for this work. Nonetheless, it still provides valuable structural information for validating and refining force field models of TMA–pyridine interactions, which shall be a strong foundation for further parametrization of the cation−π interaction in FFMD simulations.

### FFMD Simulations and Trajectory Analysis

Classical FFMD simulations were performed for two chemically distinct systems, each consisting of 40 N­(Me)_4_^+^ cations neutralized by 40 chloride anions, 1110 TIP3P water molecules, and 40 molecules of an aromatic compound (pyridine or phenol). Additionally, we explored the use of the TIP4P^31^ and ECCw2024 ^32^ water models, as discussed in the Supporting Information. The concentrations of TMACl and the aromatic compounds of 2 min were chosen to align with prior neutron scattering experimental data and to facilitate predictions for future neutron diffraction experiments.

Systems were run using GROMACS 2022,^33^ employing either the CHARMM36 ^34^ or prosECCo75 ^13^ force fields. prosECCo75 is based on CHARMM36 but differs by incorporating the electronic continuum correction (ECC) for charged moieties, which accounts for electronic polarizability by uniformly scaling the charges of all charged species by a factor of 0.75. Parameters for neutral molecules such as pyridine, phenol, and water remain unchanged from CHARMM36. Parameters for pyridine, phenol, and N­(Me)_4_^+^ in CHARMM36 were generated using CGenFF,^35^ while the prosECCo75 parameters for N­(Me)_4_^+^ were adopted from ref. 36, who found that it was not the redistribution of partial atomic charges on TMA, but rather the reduction of its overall ionic charge from +1.0 to +0.75 that improved agreement with neutron scattering data and *ab initio* calculations. When transferring from CHARMM36 to prosECCo75, the partial atomic charges of TMA differ as follows: the hydrogens of TMA have partial charges that are 0.02 units lower, the methyl carbon charges remain unchanged, and the nitrogen atom has a 0.01 unit lower charge. For clarity, the partial charges for TMA used in prosECCo75 are listed in Table 1. Chloride ions were modeled using the standard CL type^34^ (charge = −1) in CHARMM36 and the CL_2s type^37^ (charge = −0.75) in prosECCo75.

**Table 1:** Atom (Atom Type) Partial Charges of TMA

Model	N (NTL)	C (CTL5)	H (HL)	Overall charge
CHARMM	–0.60	–0.35	0.25	+1.00
prosECCo75	–0.61	–0.35	0.23	+0.75

Simulations were conducted in the isothermal–isobaric (NPT) ensemble using GROMACS 2022. The system temperature was maintained at 298 K using a V-rescale thermostat^38^ with a 1 ps coupling constant, while pressure was kept at 1 bar with a C-rescale barostat^39^ and a 5 ps coupling constant. van der Waals interactions were treated with a cutoff of 1.2 nm, employing a force-switch from 1.0 nm, and the Verlet cutoff scheme^40^ was used for neighbor searching. Long-range Coulomb interactions were accounted for using the particle mesh Ewald (PME) method, with a cutoff of 1.2 nm.^33^

The trajectories obtained from the FFMD simulations were analyzed using in-house-developed software designed for unbiased alignment and density mapping. The methodology employed closely followed the approach described previously,^36^ with the key difference being that the present analysis specifically targeted the density distributions around pyridine instead of other molecules. While direct experimental validation was limited to comparisons with structure factors from NDIS, our molecular dynamics simulations provided a deeper and more detailed structural perspective. Density maps provide a three-dimensional representation of the density distribution around a central motif. For example, to illustrate the preference for binding of TMA to pyridine, we calculated a density map showing 2.3 times the bulk density of carbons of TMA around pyridine. Although density maps cannot be directly validated through experimental measurements, they provide valuable insights into the strength and geometric characteristics of cation−π interactions. Moreover, these maps serve as an excellent benchmark for comparison with future *ab initio* molecular dynamics (AIMD), underscoring their potential in refining and validating force field models for cation−π interactions.

Visualization of simulation trajectories, density maps, and the calculation of radial distribution functions was conducted using the Visual Molecular Dynamics (VMD) software.^41^ This ensured an accurate representation and interpretation of the molecular systems, combining quantitative analysis with intuitive graphical outputs.

## Results and Discussion

From FFMD simulations, we extract the H_TMA_–H_W_ structural correlations in real (*R*) space and observe the impact of ECC thereon. Since *Q*-space and *R*-space are different representations of the same solution structure, the effects are reflected differently in each of them. In real space, this manifests as a subtle change in the radial distribution function (RDF) over a broad range of *R* values. In contrast, in *Q*-space, the same structural difference appears as a significant change at low *Q* values. Experimentally, it is challenging to measure all the low *Q* data due to the limitations in detector positioning, specifically their proximity to the direct beam passing through the sample. Obtaining these low *Q* data is essential for a fair comparison in *R*-space. Thus, *Q*-space data provide a more natural basis for comparing experimental and simulation results.

To assess the statistical accuracy of FFMD predictions against experimental data, Gaussian process regression (GPR) was applied to the experimental scattering data to estimate the mean and variance of the structure factor distribution. GPR, which is a nonparametric Bayesian method, provides a rigorous framework for quantifying the statistical properties of unknown functions given noisy observations,^42^ offering a robust way to evaluate model fits by accounting for both data trends and uncertainty. Here, we estimated the experimental uncertainty bounds using GPR and subsequently compared the CHARMM36 and prosECCo75 models to this distribution in Figure 2. A squared-exponential kernel with a white noise term was employed, using the GaussianProcessRegressor software from scikit-learn^43^ for regression and hyperparameter tuning.

**Figure 2:**
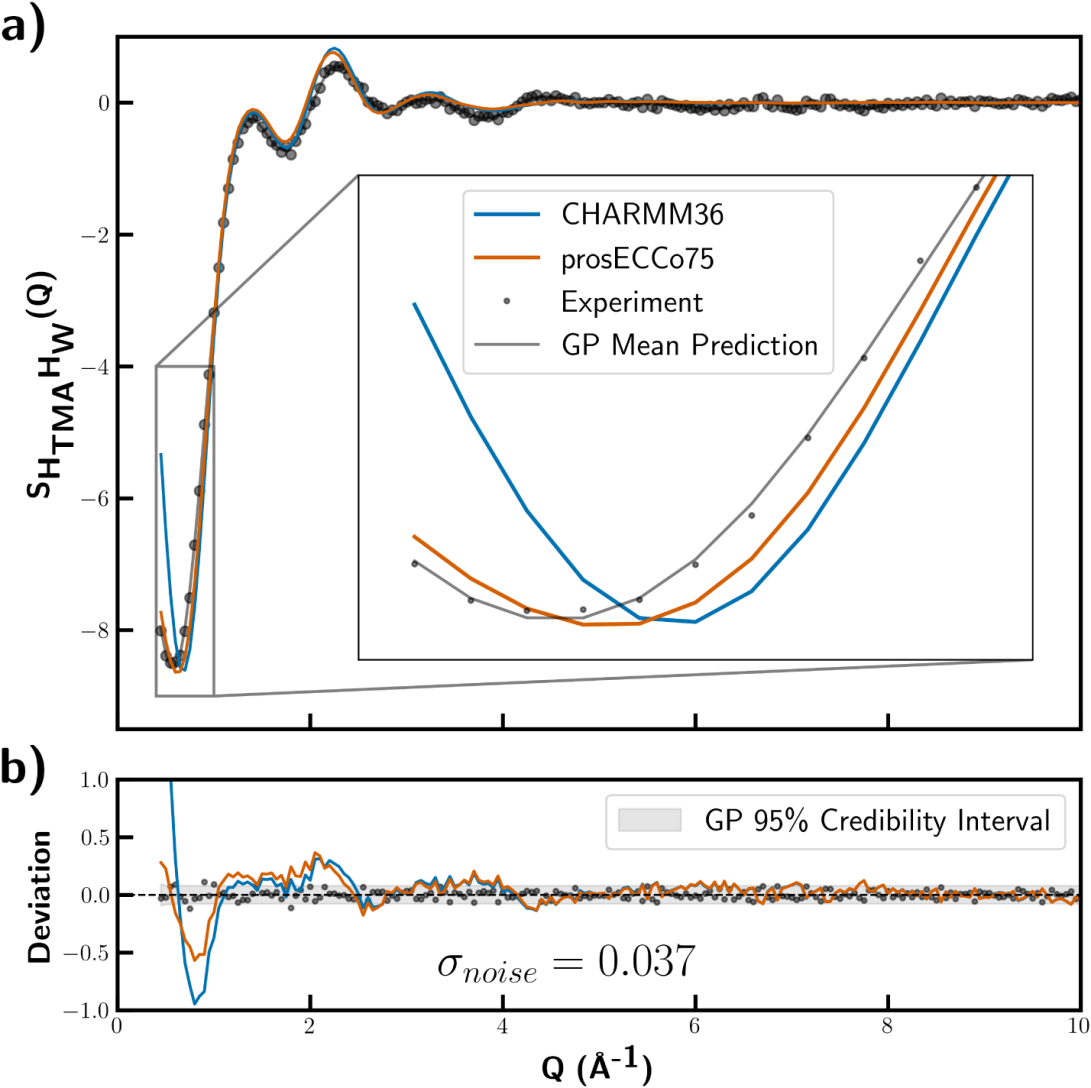
(a) Experimentally obtained $S_{H_{\mathrm{TMA}}H_{W}}\left(Q\right)$ (gray markers) compared to atomic correlation functions calculated from FFMD simulations (red/green lines) alongside the GP mean prediction (gray line). (b) Deviations of the experimental data and FFMD models from the GP mean with noise estimation (gray confidence interval).

A comparison between the GPR distribution and FFMD model predictions shows that the models significantly deviate from the experiment at *Q* < 2.5 Å^–1^. Furthermore, the difference between the CHARMM36 and prosECCo75 FFMD data becomes particularly pronounced in this low-*Q* region (Figure 2), with the latter model agreeing much better with the experiment. This result suggests that charge scaling significantly improves the description of long-range density correlations in TMA–pyridine systems. Lastly, the GPR-predicted noise on the experimental data (σ_noise_ ∼ 0.037) provides insight into whether further refinement of force field parameters may yield improved quality-of-fits to the structure. In this instance, the experimental target exceeds the σ_noise_ ∼ 0.005 precision threshold recommended for direct force field optimization to neutron scattering data,^44^ suggesting that additional experimental targets would be beneficial for further optimizing TMA–pyridine force fields.

Even though prosECCo75 and CHARMM36 differ only in their treatment of charged species, the simulations of the TMACl–pyridine system reveal stark contrasts in the structural behavior of the two force fields. To explore these differences, we calculated density maps from the simulations to visualize the geometric preferences of these interactions. Specifically, the maps represent the spatial density of TMA carbons around the atoms of the six-membered aromatic ring. The density map calculated from the trajectory using CHARMM36 FF (Figure 3a) exhibits a “headphone-like” distribution, where the density wraps around the electronegative atom of the aromatic molecule. The prosECCo75 density map (Figure 3b) displays a stronger face-on interaction, as expected for a cation−π interaction. Thanks to the improved structural agreement achieved by prosECCo75, these simulations also allow us to predict quantitatively the correlation behavior between H_TMA_ and H_Py_, as illustrated in Figure 3c. These plots show even more clearly how prosECCo75 predicts cation−π interactions stronger than those of CHARMM36. Additional neutron scattering experiments on aqueous solutions of cation−π complexes, planned with isotopic substitutions on the nonexchangable hydrogens, will directly test these predictions. Finally, note that a direct consequence of the strengthening of cation−π interactions upon moving from CHARMM36 to prosECCo75 is an increased presence of the neutralizing chloride anions in the vicinity of the aromatic molecules (see Figure S4).

**Figure 3:**
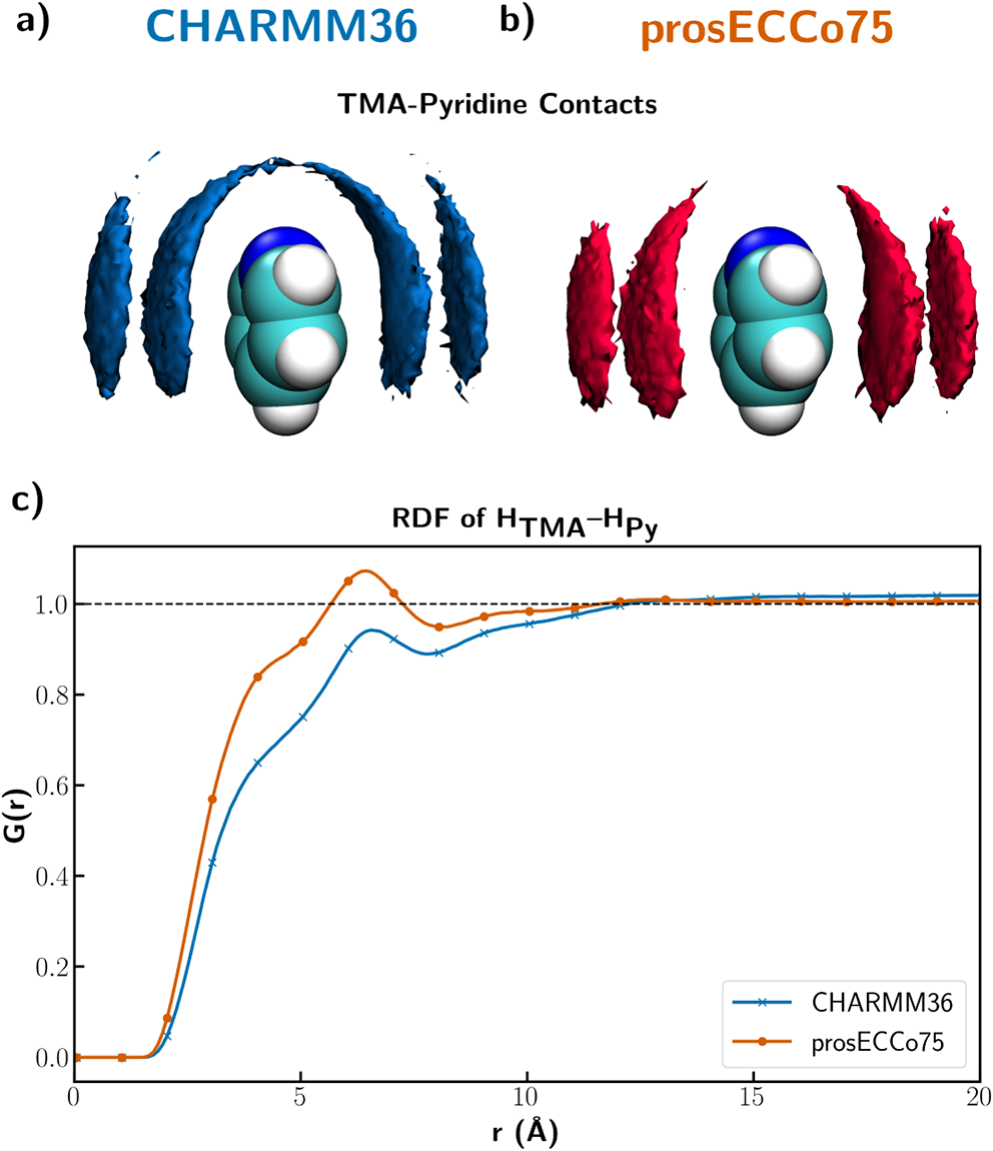
(a) and (b) Density maps of carbons on TMA around pyridine for CHARMM36 in (a) and for prosECCo75 FF in (b) (contour level 2.3 times the bulk density). (c) Radial distribution function between hydrogens on TMA and hydrogens on pyridine.

Pyridine is infinitely miscible with water at room temperature, making it an excellent candidate for NDIS experiments. However, in our simulations using the CHARMM36 force field, we observe significant aggregation of pyridine even at concentrations well below its solubility limit (2 m); as shown in Figure 4a,c, pyridine tends to form larger aggregates, and also pyridine–pyridine contacts are consistently more favored with CHARMM36. This observation is consistent with previous work suggesting that the CHARMM FF struggles to accurately capture cation−π interactions.^13,45,46^ prosECCo75 largely resolves this issue by effectively incorporating electronic polarizability. As demonstrated by the simulation snapshot and corresponding density maps of twice the bulk density of pyridine–pyridine contacts (Figure 4b,d), prosECCo75 significantly reduces the artificial aggregation observed with CHARMM36. This reduction in pyridine–pyridine contacts is mostly due to enhanced competition with pyridine–TMA cation−π interactions.

**Figure 4:**
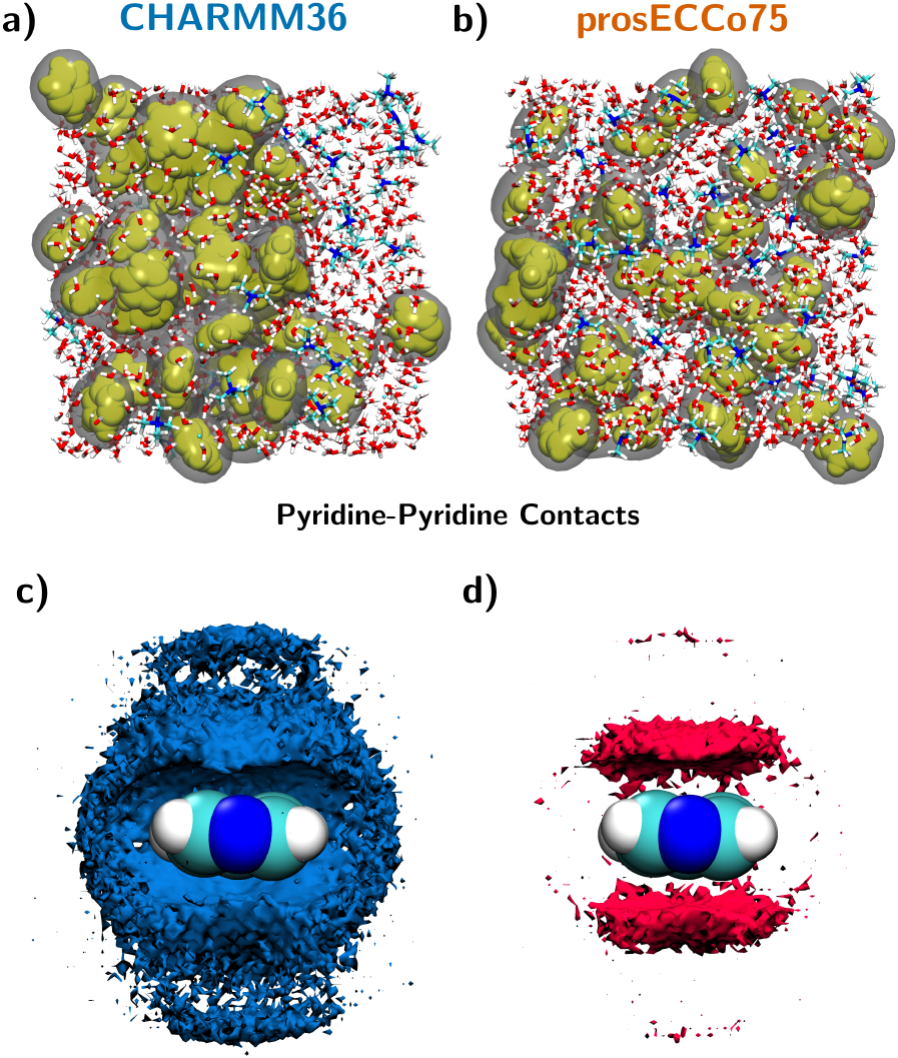
(a) and (b) Snapshots from FFMD simulations of the 2 m TMACl–pyridine system using (a) CHARMM36 and (b) prosECCo75 force fields. Pyridine molecules are shown in yellow, TMACl and water in licorice representation, and gray isosurfaces outline pyridine–pyridine contacts. (c) and (d) Density maps illustrating pyridine–pyridine interactions, represented as twice the bulk density of pyridine ring-member atoms relative to pyridine hydrogen atoms, calculated from trajectories using (c) CHARMM36 and (d) prosECCo75 force fields.

Although prosECCo75 achieves significantly closer agreement with structure factors from NDIS experiments than CHARMM36, this marks only the beginning of advancing the description of cation−π interactions in FFMD. For example, further refinement of the charge-scaled description of cation−π interactions could be explored using machine learning accelerated force field optimization to NDIS data^47,48^ or to more fundamental simulation methods such as AIMD. Finally, further work is required to investigate a wider range of biologically relevant cationic species, such as guanidinium, and other aromatic motifs, like phenol or indole. To this end, we have additionally performed as a first step simulations on the TMACl–phenol system, which is even more directly relevant to biological applications than pyridine since it more closely resembles the aromatic side chains of amino acid tyrosine.

The solubility of phenol in water is about 0.9 m. One might thus argue that NDIS experiments requiring for sufficient resolution molar aqueous solutions should not be feasible; nevertheless, we have found that in the presence of a 3 m solution of TMACl, the solubility of phenol increases to about 3 m. This is presumably due to the favorable TMA–phenol interaction, consistent with the use of quaternary ammonium salts in deep eutectic solvent extraction for aromatic species.^49,50^ As expected, prosECCo75 reproduces this behavior by keeping phenol dissolved (Figure 5b,d), whereas CHARMM36 exhibits more extensive phenol aggregation (Figure 5a,c). Similarly to TMA–pyridine, CHARMM36 leads to a more pronounced “headphone-like” arrangement favoring TMA interaction with the ring’s electronegative hydroxyl motif. However, for the remaining aromatic ring, the face-on cation−π interaction is significantly stronger in prosECCo75 than in CHARMM36 (Figure 6a,c). Similarly to the case of pyridine, we can use these simulations to predict the correlations between H_TMA_ and H_Ph_ (Figure 6c), which show even more clearly the strengthening of cation−π interactions upon charge scaling, to be tested by the upcoming neutron scattering measurements. These experiments will further assess the reliability and versatility of prosECCo75, the accuracy and robustness of which are grounded in the physically well-justified ECC scaling approach.

**Figure 5:**
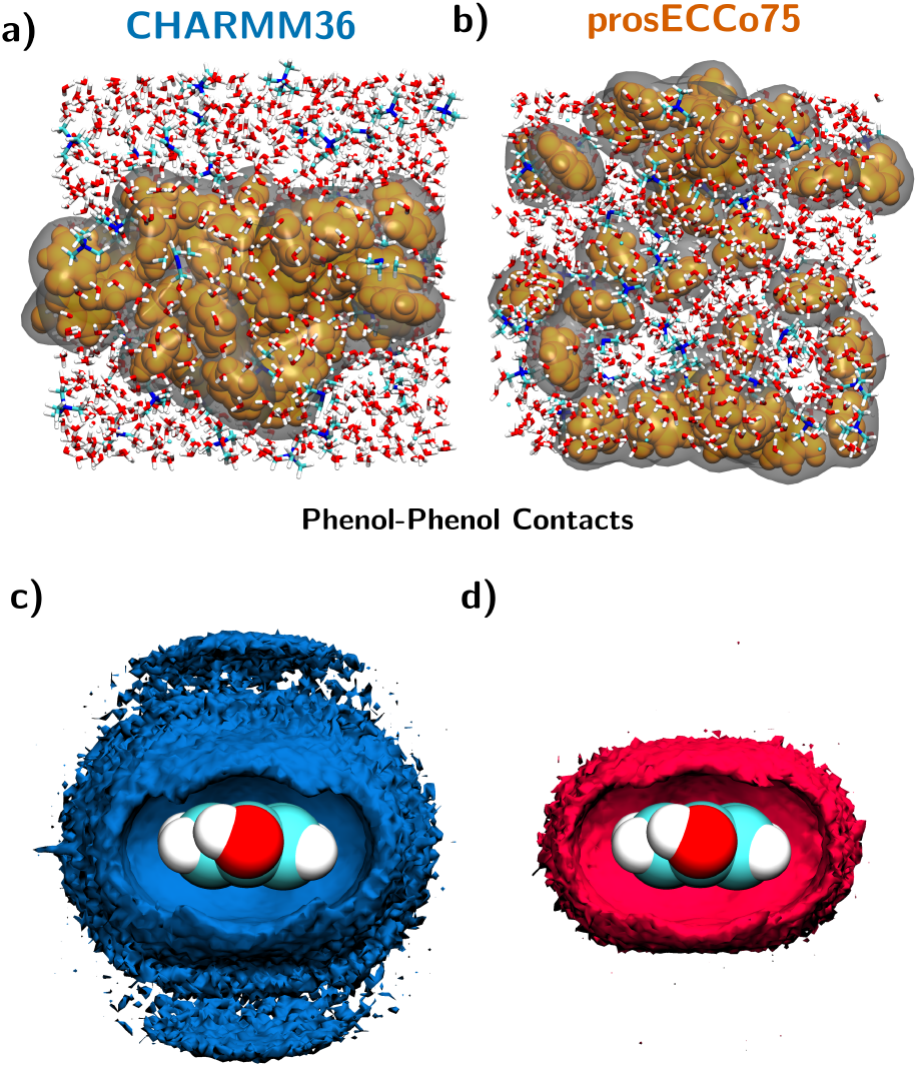
(a) and (b) Snapshots from FFMD simulations of the 2 m TMACl–phenol system using (a) CHARMM36 and (b) prosECCo75 force fields. Phenol molecules are shown in orange, TMACl and water in licorice representation, and gray isosurfaces outline phenol–phenol contacts. (c) and (d) Density maps illustrating phenol–phenol interactions, represented as regions of 3.3 times the bulk density of phenol ring-member atoms relative to phenol hydrogen atoms, calculated from trajectories using (c) CHARMM36 and (d) prosECCo75 force fields.

**Figure 6:**
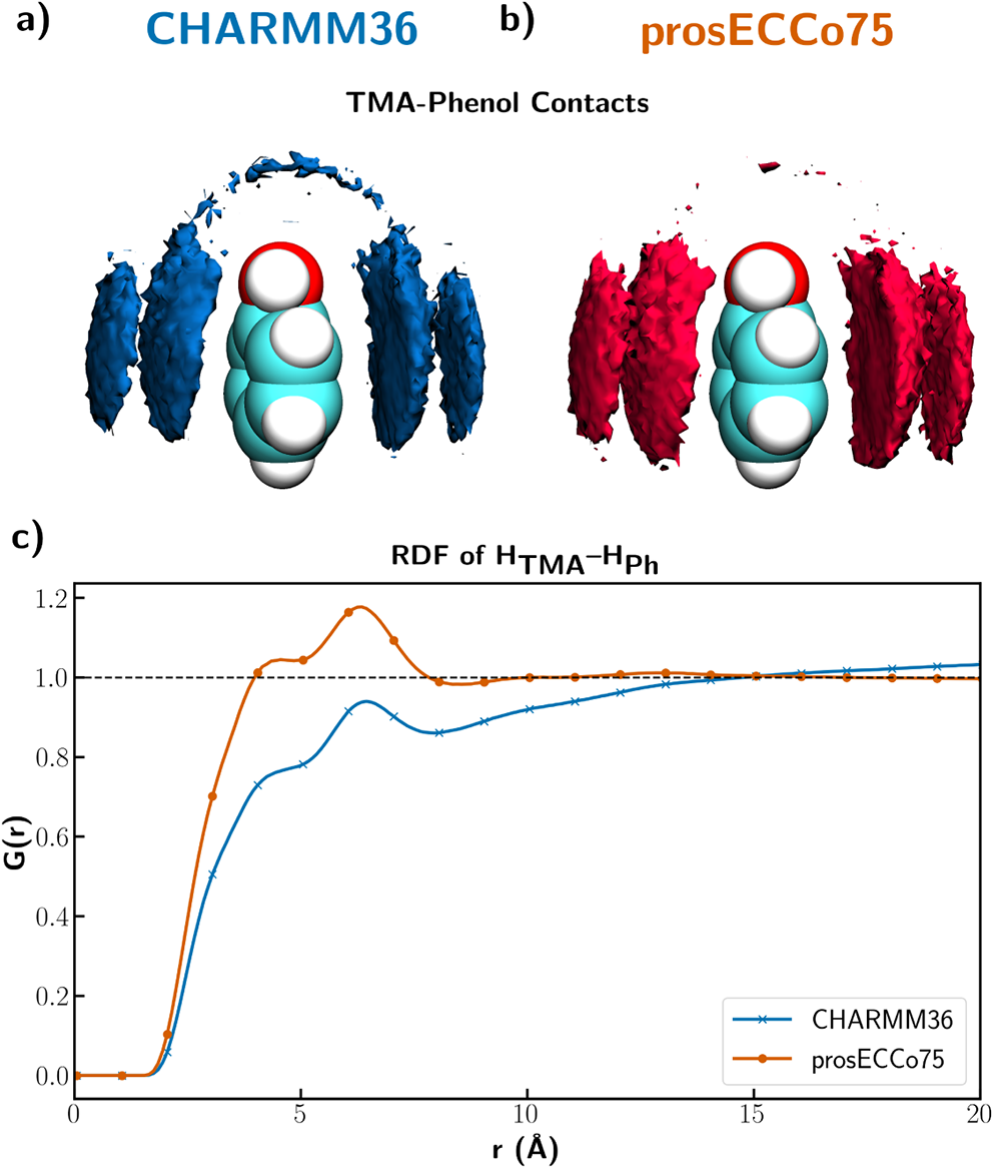
(a) and (b) Density maps of carbons on TMA around phenol for CHARMM36 in (a) and prosECCo75 FF in (b) (2.7 times the bulk density). (c) Radial distribution function between hydrogens on TMA and hydrogens on phenol. This interaction is about 50% stronger than the TMA–pyridine cation–pi interaction.

Finally, we note that the positive effect of charge scaling on the accuracy of the description of cation−π interactions involving TMA is to some extent surprising. Indeed, our previous studies have demonstrated that the effect of ECC on the strength of ion pairing in aqueous solutions diminishes with decreasing charge density, becoming rather weak for ions like TMA.^51^ The present work shows that this is not true for cation−π interactions, where accounting for electronic polarization via charge scaling helps to establish the right balance between electrostatic and hydrophobic forces.

## Conclusions

Our experiments and simulations indicate that effectively incorporating electronic polarization via charge scaling in the prosECCo75 model provides results for cation−π interactions involving tetramethylammonium and pyridine that are in closer agreement with neutron scattering data compared with results obtained using the original CHARMM36 force field. prosECCo75 yields a stronger face-on interaction (compared to CHARMM36) of TMA binding to the aromatic ring. This is consistent with the expected geometry of the cation−π interaction. By reducing the net charge of the ion, ECC appears to lower its hydration penalty enough to facilitate a more pronounced interaction with the aromatic π-system in water, which also removes the pyridine aggregation artifact and the resulting low solubility caused by the use of CHARMM36.

Additionally, based on our simulations, we made a specific prediction for the structure of the aqueous TMA–phenol complex, to be tested by future neutron scattering experiments. Phenol is a particularly relevant aromatic molecule for biological contexts, as it mirrors the side chain of tyrosine. Our simulations show that prosECCo75 alters, compared to CHARMM36, the overall behavior of these cation−π interactions in a manner similar to TMA–pyridine, with the effect about twice as strong with phenol as it is for pyridine. Specifically, while CHARMM36 exhibits artifactual phenol aggregation, prosECCo75 predicts a stronger cation−π interaction and yields reduced phenol aggregation. This is also consistent with the known ability of quaternary ammonium ions to enhance the solubility of aromatic species. The consistency of the prosECCo75 prediction for both TMA–pyridine and TMA–phenol interactions suggests that the present charge-scaling approach may be widely applicable to a broader class of cation−π motifs commonly found in biomolecules, offering a promising strategy for improving the accuracy of molecular dynamics simulations for these complex systems.

## Supplementary Material



## Data Availability

The NDIS experimental data and FFMD data supporting this study are available in the https://github.com/cervthecoder/cervenka_tma GitHub repository. Due to storage limitations, trajectory files are not included in the repository but can be made available upon reasonable request.

## Notes

Table S1: partial charges and atom types of pyridine; Table S2: partial charges and atom types for phenol; Figure S1: *G*(*r*) correlation of H_W_–H_TMA_ in *r*-space; Figure S2: structure factor comparison between experiment, TIP3P and TIP4P water models, Figure S3: *G*(*r*) correlation of H_TMA_–H_Ph_ in *r*-space; Figure S4: radial distribution functions comparing CHARMM36 and prosECCo75 force fields; Figure S5: pyridine molecule with atoms corresponding to Table S1; Figure S6: phenol molecule with atoms corresponding to Table S2 (PDF)

## References

[1] Ooi S. K. T., Qiu C., Bernstein E., Li K., Jia D., Yang Z., Erdjument-Bromage H., Tempst P., Lin S.-P., Allis C. D. (2007). DNMT3L connects unmethylated lysine 4 of histone
H3 to de novomethylation of DNA. Nature.

[2] Gao J., Chou L. W., Auerbach A. (1993). The Nature of Cation-Pi Binding:
Interactions between Tetramethylammonium Ion and Benzene in Aqueous
Solution. Biophys. J..

[3] Cheng J., Goldstein R., Gershenson A., Stec B., Roberts M. F. (2013). The Cation-
Box Is a Specific Phosphatidylcholine Membrane Targeting Motif *. J. Biol. Chem..

[4] Bellamy H. D., Lim L. W., Mathews F. S., Dunham W. R. (1989). Studies ofCrystalline
Trimethylamine Dehydrogenase in Three Oxidation States and in the
Presence of Substrate and Inhibitor. J. Biol.
Chem..

[5] Dougherty D. A. (1996). Cation-Interactions
in Chemistry and Biology: A New View of Benzene, Phe, Tyr, and Trp. Science.

[6] Tsuzuki S., Yoshida M., Uchimaru T., Mikami M. (2001). The Origin
of the Cation/
Interaction: The Significant Importance of the Induction in Li+ and
Na+ Complexes. J. Phys. Chem. A.

[7] Khan H. M., MacKerell A. D. J., Reuter N. (2019). Cation- Interactions between Methylated
Ammonium Groups and Tryptophan in the CHARMM36 Additive Force Field. J. Chem. Theory Comput..

[8] Turupcu A., Tirado-Rives J., Jorgensen W. L. (2020). Explicit
Representation of Cation-
Interactions in Force Fields with 1/R4 Nonbonded Terms. J. Chem. Theory Comput..

[9] Lin F.-Y., MacKerell D., MacKerell J. (2020). Improved Modeling
of Cation-π
and Anion-Ring Interactions Using the Drude Polarizable Empirical
Force Field for Proteins. J. Comput. Chem..

[10] Felder C., Jiang H.-L., Zhu W.-L., Chen K.-X., Silman I., Botti S. A., Sussman J. L. (2001). Quantum/Classical Mechanical Comparison
of Cation-Interactions between Tetramethylammonium and Benzene. J.Phys. Chem. A.

[11] Teng X., Yu W., MacKerell A. D. (2025). Computationally Efficient Polarizable MD Simulations:
A Simple Water Model for the Classical Drude Oscillator Polarizable
Force Field. J. Phys. Chem. Lett..

[12] Leontyev I. V., Stuchebrukhov A. A. (2010). Electronic
Continuum Model for Molecular Dynamics Simulations
of Biological Molecules. J.Chem. Theory Comput..

[13] Nencini R., Tempra C., Biriukov D., Riopedre-Fernandez M., Cruces Chamorro V., Polak J., Mason P. E., Ondo D., Heyda J., Ollila O. H. S. (2024). Effective Inclusion
of Electronic PolarizationImproves the Description of Electrostatic
Interactions: TheprosECCo75 Biomolecular Force Field. J. Chem. Theory Comput..

[14] MacKerell A. D. J., Bashford D., Bellott M., Dunbrack R. L. J., Evanseck J. D., Field M. J., Fischer S., Gao J., Guo H., Ha S. (1998). All-Atom Empirical Potential for Molecular
Modeling
and Dynamics Studies of Proteins. J. Phys. Chem.
B.

[15] Jorge M., Barrera M. C., Milne A. W., Ringrose C., Cole D. J. (2023). What Is
the Optimal Dipole Moment for Nonpolarizable Models of Liquids?. J. Chem. Theory Comput..

[16] Jorge M. (2024). Theoretically
Grounded Approaches to Account for Polarization Effects in Fixed-Charge
Force Fields. J. Chem. Phys..

[17] McFegan L., Juhasz A., Marton P., Hórvölgyi Z., Jedlovszky-Hajdu A., Hantal G., Jedlovszky P. (2023). Surface Affinity
of Tetramethylammonium Iodide inAqueous Solutions: A Combined Experimental
and Computer Simulation Study. J. Phys. Chem.
B.

[18] de
Jong P. H. K., Neilson G. W. (1996). Structural Studies of Ionic Solutions
underCritical Conditions. J. Phys.: Condens.
Matter.

[19] Mason P. E., Neilson G. W., Dempsey C. E., Brady J. W. (2006). NeutronDiffraction
and Simulation Studies of CsNO3 and Cs2CO3 Solutions. J. Am. Chem. Soc..

[20] Mason P. E., Ansell S., Neilson G. W. (2006). Neutron Diffraction Studies of Electrolytes
in Null Water: A Direct Determination of the First HydrationZone of
Ions. J. Phys.: condens. Matter.

[21] Neilson G. W., Mason P. E., Ramos S., Sullivan D. (2001). Neutronand X-Ray Scattering
Studies of Hydration in Aqueous Solutions. Philos.
Trans. R. Soc. London, A.

[22] Turner J. A. S., Soper A. K., Finney J. L. (1990). A neutron-diffraction study of tetramethylammonium
chloride in aqueous solution. Mol. Phys..

[23] Mason P. E., Neilson G. W., Dempsey C. E., Price D. L., Saboungi M.-L., Brady J. W. (2010). Observation of Pyridine
Aggregation in Aqueous SolutionUsing
Neutron Scattering Experiments and MD Simulations. J. Phys. Chem. B.

[24] Turner J., Soper A., Finney J. (1992). Water Structure in Aqueous Solutions
ofTetramethylammonium Chloride. Mol. Phys..

[25] Turner J. Z., Soper A. K., Finney J. L. (1995). Ionic versus
Apolar Behavior of theTetramethylammonium
Ion in Water. J. Chem. Phys..

[26] Nilsson E. J., Alfredsson V., Bowron D. T., Edler K. J. (2016). A Neutron Scattering
and Modelling Study of Aqueous Solutions of Tetramethylammoniumand
Tetrapropylammonium Bromide. Phys. Chem. Chem.
Phys..

[27] Fischer H., Cuello G., Palleau P., Feltin D., Barnes A., Badyal Y., Simonson J. (2002). D4c: A Very
High Precision Diffractometer
for Disordered Materials. Appl. Phys. A: Mater.
Sci. Process..

[28] Mason P. E., Ansell H. E., Neilson G. W., Rempe S. (2015). Towards a Fuller Understanding
of Protein–Lipid Interactions. J. Phys.
Chem. B.

[29] Herdman G. J., Neilson G. W. (1992). Ferric Ion (Fe­(III)) Coordination inConcentrated Aqueous
Electrolyte Solutions. J. Phys.: condens. Matter.

[30] Enderby J. E., Richards R. E., Williams R. J. P. (1997). Neutron
and X-ray Scattering from
Aqueous Solutions. Proc. R. Soc. London A.

[31] Abascal J. L. F., Vega C. (2005). A General Purpose Model for the Condensed
Phases of
Water: TIP4P/2005. J. Chem. Phys..

[32] Cruces
Chamorro V., Jungwirth P., Martinez-Seara H. (2024). Building Water
Models Compatible with Charge Scaling Molecular Dynamics. J. Phys. Chemlett..

[33] Abraham M. J., Murtola T., Schulz R., Pall S., Smith J. C., Hess B., Lindahl E. (2015). GROMACS: High Performance
Molecular
Simulations through Multi-Level Parallelism from Laptops to Supercomputers. SoftwareX.

[34] Huang J., MacKerell D., MacKerell J. (2013). CHARMM36 All-Atom
Additive ProteinForce
Field: Validation Based on Comparison to NMR Data. J. Comput. Chem..

[35] Vanommeslaeghe K., Hatcher E., Acharya C., Kundu S., Zhong S., Shim J., Darian E., Guvench O., Lopes P., Vorobyov I. (2010). CHARMMGeneral
Force Field (CGenFF): A Force
Field for Drug-like Molecules Compatible with the CHARMM All-Atom
Additive Biological Force Fields. J. Comput.
Chem..

[36] Mason P. E., Martinek T., Fabian B., Vazdar M., Jungwirth P., Tichacek O., Duboué-Dijon E., Martinez-Seara H. (2024). Hydration
of Biologically Relevant Tetramethylammonium Cation by Neutron Scattering
and MolecularDynamics. Phys. Chem. Chem. Phys..

[37] Pluharova E., Fischer H. E., Mason P. E., Jungwirth P. (2014). Hydration
of the Chloride Ion in Concentrated Aqueous Solutions Using Neutron
Scattering andMolecular Dynamics. Mol. Phys..

[38] Bussi G., Donadio D., Parrinello M. (2007). Canonical
Sampling through Velocity
Rescaling. J. Chem. Phys..

[39] Bussi G., Parrinello M. (2008). Stochastic Thermostats: Comparison of Local and Global
Schemes. Comput. Phys. Commun..

[40] Pall S., Hess B. (2013). A Flexible Algorithm
for Calculating Pair Interactions on SIMD Architectures. Comput. Phys. Commun..

[41] Humphrey W., Dalke A., Schulten K. (1996). VMD: Visual Molecular
Dynamics. J. Mol. Graphics.

[42] Rasmussen, C. E. ; Williams, C. K. I. Gaussian Processes for MachineLearning; The MIT Press, 2005.

[43] Pedregosa F., Varoquaux G., Gramfort A., Michel V., Thirion B., Grisel O., Blondel M., Prettenhofer P., Weiss R., Dubourg V. (2011). Scikit-learn: Machine
Learning in Python. J. Mach.Learn. Res..

[44] Shanks B. L., Sullivan H. W., Hoepfner M. P. (2024). Bayesian
Analysis Revealsthe Key
to Extracting Pair Potentials from Neutron Scattering Data. J. Phys. Chem. Lett..

[45] Yoo J., Aksimentiev A. (2018). New Tricks for Old Dogs: Improving the Accuracy of
Biomolecular Force Fields by Pair-Specific Corrections to Non-BondedInteractions. Phys. Chem. Chem. Phys..

[46] Croitoru A., Park S.-J., Kumar A., Lee J., Im W., MacKerell A. D. J., Aleksandrov A. (2021). Additive CHARMM36
Force Field for
Nonstandard AminoAcids. J. Chem. Theory Comput..

[47] Shanks B. L., Sullivan H. W., Shazed A. R., Hoepfner M. P. (2024). Accelerated Bayesian
Inference for Molecular Simulations Using Local Gaussian Process Surrogate
Models. J. Chem. Theory Comput..

[48] Shanks B. L., Potoff J. J., Hoepfner M. P. (2022). Transferable
Force Fields from Experimental
Scattering Data with Machine Learning Assisted Structure Refinement. J. Phys. Chem. Lett..

[49] de
Almeida Pontes P. V., Ayumi Shiwaku I., Maximo G. J., CaldasBatista E. A. (2021). Choline
Chloride-Based Deep Eutectic Solvents as Potential Solvent for Extraction
of Phenolic Compounds from Olive Leaves: Extraction Optimization and
Solvent Characterization. Food Chem..

[50] García A., Rodríguez-Juan E., Rodríguez-Gutiérrez G., Rios J. J., Fernandez-Bolaños J. (2016). Extraction
of Phenolic
Compounds from Virgin Olive Oilby Deep Eutectic Solvents (DESs). Food Chem..

[51] Nguyen N. L. L., Tichacek O., Jungwirth P., Martinez-Seara H., Mason P. E., Duboué-Dijon E. (2025). Ion pairing
in aqueous tetramethylammonium–acetate
solutions by neutron scattering and molecular dynamics simulations. Phys. Chem. Chem. Phys..

