# Supplementary material for: Cation−π Interactions in Biomolecular Contexts by Neutron Scattering and Molecular Dynamics: A Case Study of the Tetramethylammonium Cation
Source: J Phys Chem B. 2025 Jun 27;129(27):6911–8. doi: 10.1021/acs.jpcb.5c02001 (PMC12257538; doi:10.1021/acs.jpcb.5c02001)
Supplement: Supplementary file 1 [file jp5c02001_si_001.pdf]

Supporting Information: Cation-π Interactions in Biomolecular Contexts by Neutron Scattering and Molecular Dynamics: Case Study of the Tetramethylammonium Cation

Matej Cervenka[a], Brennon L. Shanks[a], Philip E. Mason[a], and Pavel Jungwirth[a*]

[a]*Institute of Organic Chemistry and Biochemistry of the Czech Academy of Sciences, Flemingovo nm. 2, 166 10 Prague 6, Czech Republic*

E-mail: philip.mason@uochb.cas.cz,pavel.jungwirth@uochb.cas.cz

Water Models Comparison

prosECCo75 was initially developed with the TIP3P water model,[S1] due to its compatibility with CHARMM, as all neutral species remain unchanged between the two. However, we sought to investigate whether the choice of water model significantly impacts the primary conclusion that charge scaling results in a better representation of liquid phase structure when compared to experimental data. To this end, we repeated both the non-scaled and scaled charge calculations using the TIP4P[S2] and ECCw2O24[S3] water models, both of which are 4-site models, and present the comparison here.

The structural correlations between H_W-H_{TMA} in TMACl-pyridine system for TIP3P and the 4-site water models are illustrated in r-space in Fig. S1 and in Q-space in Fig. S2. Clearly, applying charge scaling to systems with either TIP3P, TIP4P or ECCw2024 significantly improves the fit to the experimental structure factor compared to their non-scaled counterparts. This improvement is especially significant at low-Q, indicating a better representation of clustering. Consistent with previous studies, the prosECCo75 model with TIP3P water continues to provide the best overall fit to the experimental structure factor.

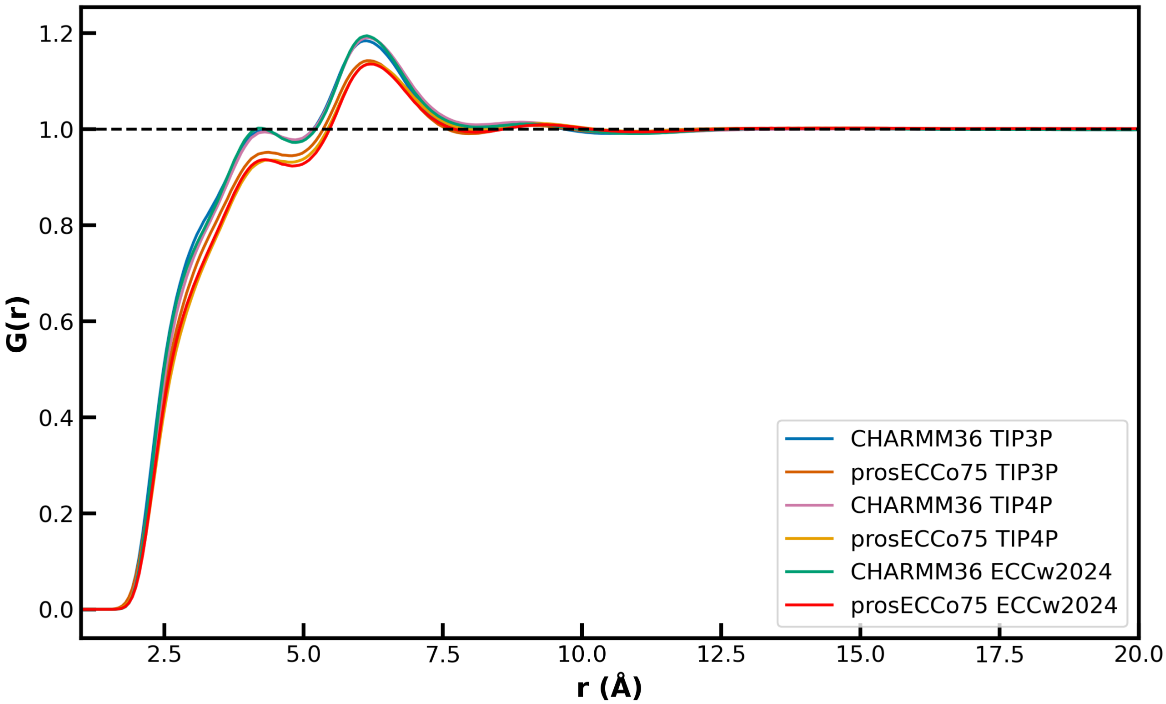

Figure S1: G(r) correlation of H_W-H_{TMA} in r-space.

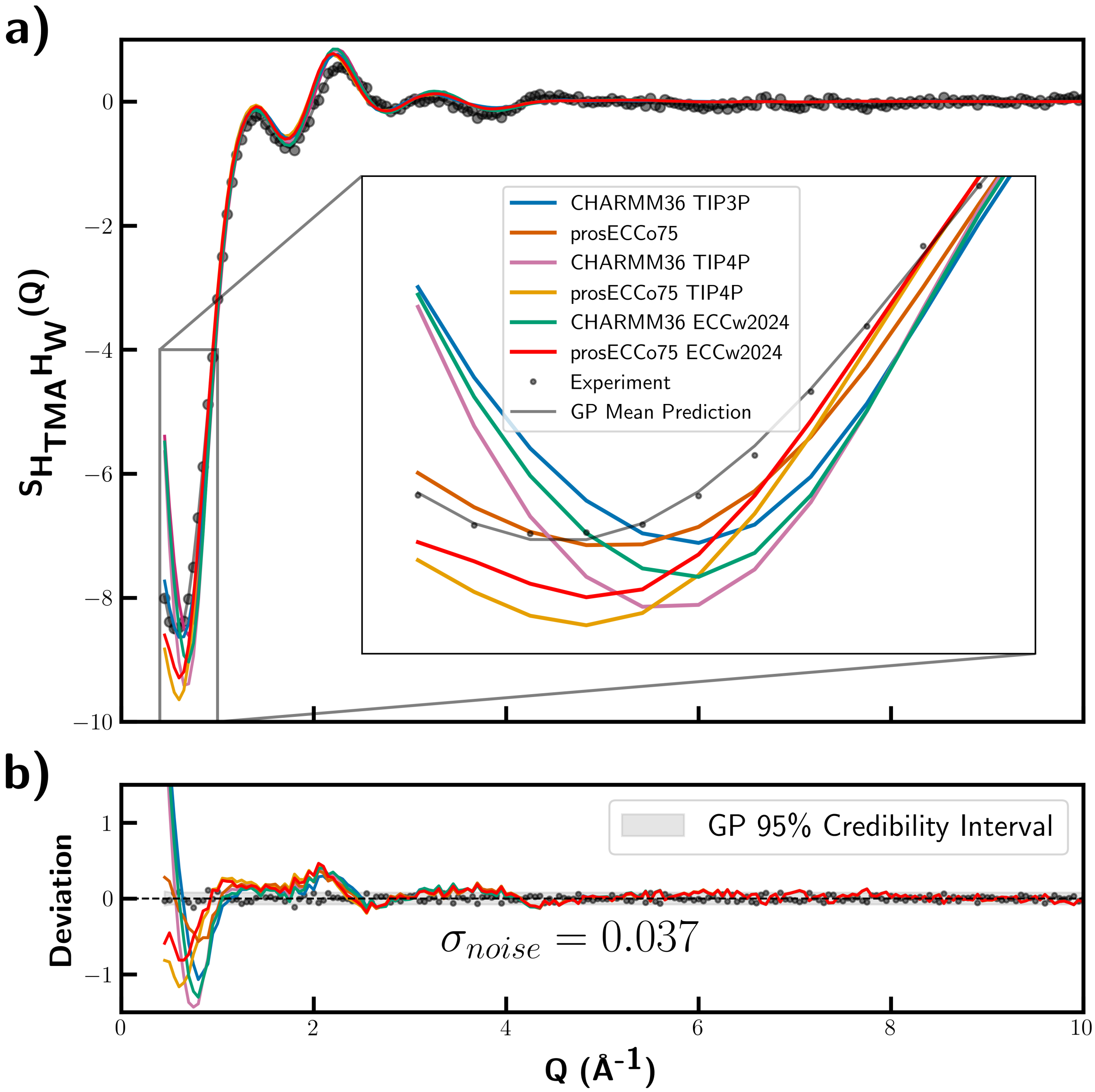

Figure S2: Structure factor comparison between experiment, TIP3P and TIP4P water models. (a) Experimentally measured $\Delta\Delta S_{\mathrm{H_{non}}}(Q) = 114.6 \cdot \left(S_{\mathrm{H_{TMA}H_W}}(Q) - 1\right)$ (gray markers) compared against FFMD simulations using CHARMM36 TIP3P (blue), prosECCo75 TIP3P (red), CHARMM36 TIP4P (purple), prosECCo75 TIP4P (orange), CHARMM36 ECCw2024 (green), prosECCo75 ECCw2024 (dark red). The Gaussianprocess (GP) mean prediction is shown as a gray line. (b) Deviations of both the experimental data (gray markers) and the FFMD simulations (colored lines) from the GP mean, with the shaded gray region indicating the GP 95% credibility interval. The estimated noise level is $\sigma_{\mathrm{noise}} = 0.037$.

prosECCo75 was primarily developed using the TIP3P water model,[S1] so we expect TMAphenol predictions with this model to be the most reliable. Encouragingly, simulations with TIP4P

water produce nearly identical RDFs in shape and peak position (Fig. S3). The only notable difference is a slight increase in peak height, which indicates a higher average number of neighbors in TIP4P water, an effect that is even more pronounced with ECCw2024.

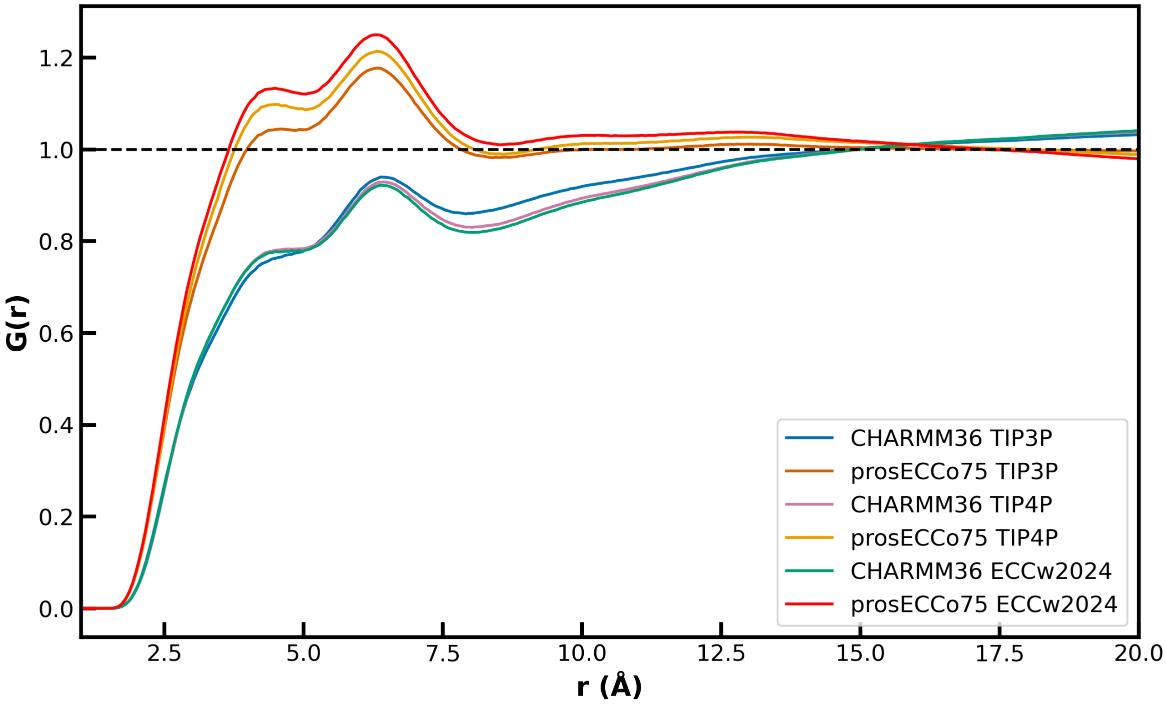

Figure S3: G(r) correlation of H_{TMA}-H_{Ph} in r-space.

Role of Chloride Anions

It is important to mention the role of chloride ions in the TMACl-pyridine and TMACl-phenol systems. As noted in the main text, aromatic molecules have significant quadrupole moment,[S4] which primarily interacts with the cation but can also interact with the anion through their partially positively charged regions. Additionally, the anion tends to follow the cation to maintain local electroneutrality around the aromatic motif (Fig. S4e and S4f). However, as shown in the radial distribution functions, G(r), for the center of geometry of the aromatic motif relative to either the nitrogen atom in TMA (Fig. S4a and S4b) or the chloride ion (Fig. S4c and S4d), the interactions between TMA and aromatic molecules are considerably stronger than those involving the chloride

ion for both systems. Moreover, the prosECCo75 model increases the strength of the interaction between TMA and aromatic molecules compared to CHARMM36.

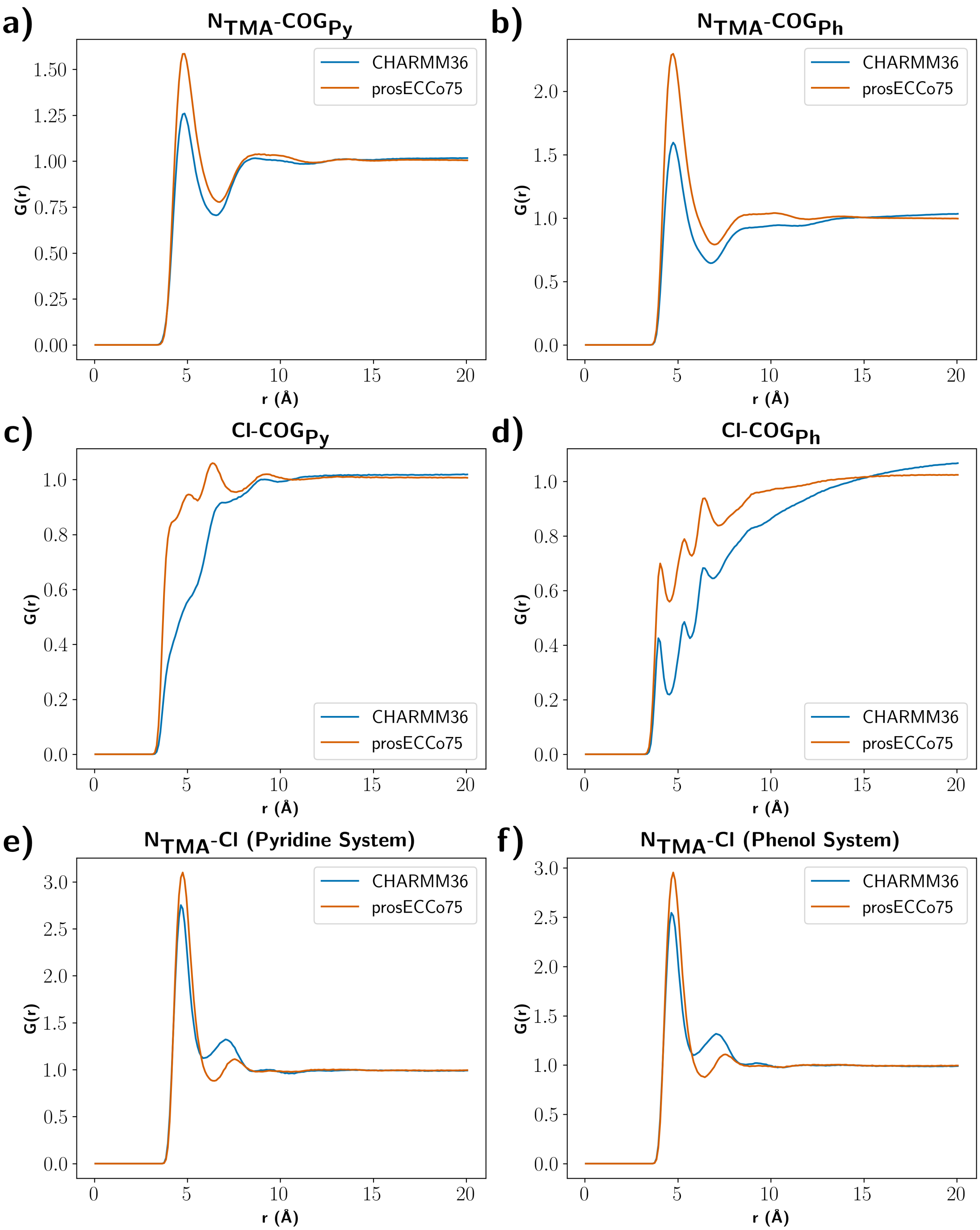

Figure S4: Radial distribution functions comparing CHARMM36 (blue line) and prosECCo75 (red line) force fields: (a) nitrogen atom of TMA relative to the center of geometry (COG) of pyridine; (b) nitrogen atom of TMA relative to the COG of phenol; (c) chloride ion relative to the COG of pyridine; (d) chloride ion relative to the COG of phenol; (e) nitrogen atom of TMA relative to chloride ion in the TMACl-Pyridine system; (f) nitrogen atom of TMA relative to chloride ion in the TMACl-Phenol system.

Force Field Parameters of Pyridine and Phenol

Below are the force field parameters (partial atomic charges and atom types) for pyridine and phenol within the CHARMM36 force field, generated using the CGenFF program.[S5] Atom names in Tables S1 and S2 correspond to the labeling shown in Figures S5 and S6, respectively.

Table S1: Partial charges and atom types of pyridine

Atom	Partial charge	Atom type
N1	−0.598	NG2R60
H1	+0.122	HGR62
H2	+0.115	HGR61
H3	+0.115	HGR61
C1	+0.182	CG2R61
C2	−0.121	CG2R61
C3	−0.113	CG2R61

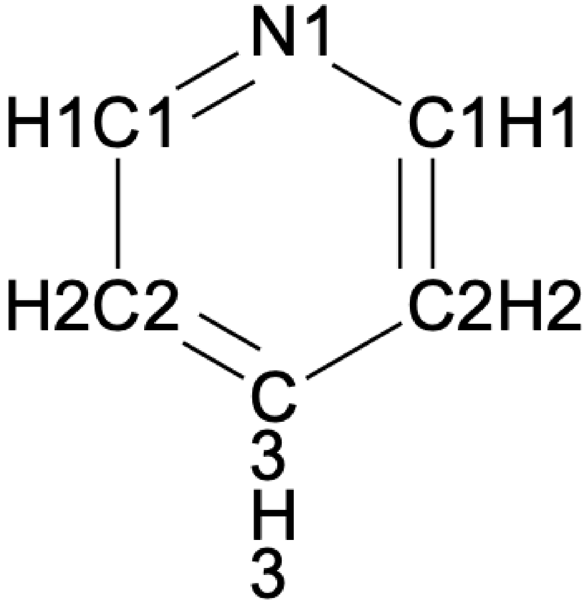

Figure S5: Pyridine molecule with atoms corresponding to Table S1

Table S2: Partial charges and atom types for phenol

Atom	Partial charge	Atom type
O1	−0.533	OG311
H1	+0.420	HGP1
H2	+0.115	HGR61
H3	+0.115	HGR61
H4	+0.115	HGR61
C1	+0.107	CG2R61
C2	−0.113	CG2R61
C3	−0.114	CG2R61
C4	−0.115	CG2R61

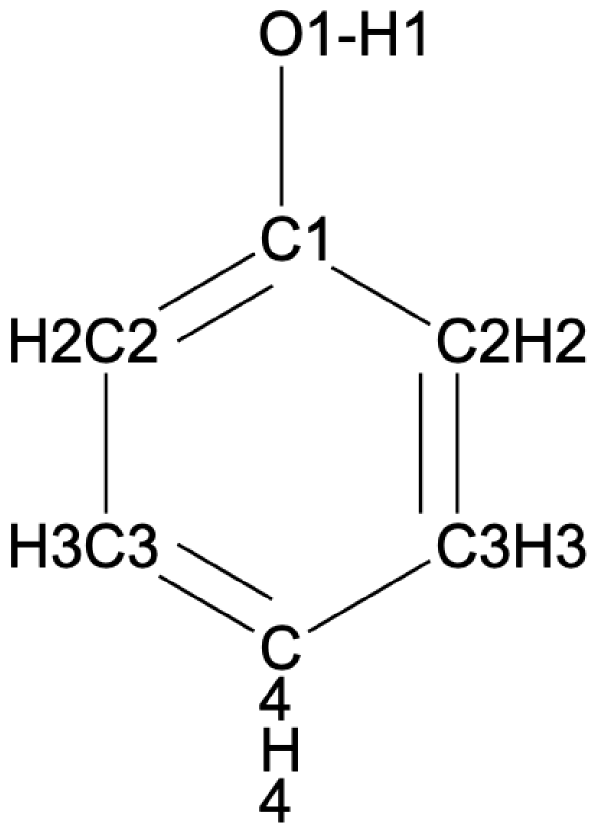

Figure S6: Phenol molecule with atoms corresponding to Table S2

References

(S1) Nencini, R.; Tempra, C.; Biriukov, D.; Riopedre-Fernandez, M.; Cruces Chamorro, V.; Polak, J.; Mason, P. E.; Ondo, D.; Heyda, J.; Ollila, O. H. S. et al. Effective Inclusion of Electronic Polarization Improves the Description of Electrostatic Interactions: The prosECCo75 Biomolecular Force Field. *J. Chem. Theory Comput.* **2024**, *20*, 7546–7559.

(S2) Abascal, J. L. F.; Vega, C. A General Purpose Model for the Condensed Phases of Water: TIP4P/2005. *J. Chem. Phys.* **2005**, *123*, 234505.

(S3) Cruces Chamorro, V.; Jungwirth, P.; Martinez-Seara, H. Building Water Models Compatible with Charge Scaling Molecular Dynamics. *J. Phys. Chem. Lett.* *15*, 2922–2928.

(S4) Dougherty, D. A. Cation- Interactions in Chemistry and Biology: A New View of Benzene, Phe, Tyr, and Trp. *Science* **1996**, *271*, 163–168.

(S5) Vanommeslaeghe, K.; Hatcher, E.; Acharya, C.; Kundu, S.; Zhong, S.; Shim, J.; Darian, E.; Guvench, O.; Lopes, P.; Vorobyov, I. et al. CHARMM General Force Field (CGenFF): A Force Field for Drug-like Molecules Compatible with the CHARMM All-Atom Additive Biological Force Fields. *J. Comput. Chem.* **2010**, *31*, 671.
